# Influence of Postharvest Temperatures on Carotenoid Biosynthesis and Phytochemicals in Mature Green Chili (*Capsicum annuum* L.)

**DOI:** 10.3390/antiox9030203

**Published:** 2020-03-01

**Authors:** Wissanee Pola, Sumiko Sugaya, Songsin Photchanachai

**Affiliations:** 1 Division of Postharvest Technology, School of Bioresources and Technology, King Mongkut’s University of Technology Thonburi, 49, Soi Thiantale 25, Bangkhuntien-Chaitale Rd., Thakham, Bangkhuntien, Bangkok 10150, Thailand; wissaneepola@gmail.com; 2 Laboratory of Pomology, Graduate School of Life and Environmental Sciences, University of Tsukuba, Ibaraki 305-8572, Japan; sugaya.sumiko.fw@u.tsukuba.ac.jp

**Keywords:** antioxidants, gene expression, incubation, intense red color, mature green chili

## Abstract

An intense red color appearance in hot chili is what industry commonly demands. The harvested mature green “Takanotsume” chili, a popular cultivar in Japan, incubated at 20 and 30 °C is investigated. At 30 °C, the chili rapidly degraded chlorophylls and obtained an intense red color, but presented an orange–red color at 20 °C. The sample showed higher carotenoid accumulations at 30 °C, along with significantly upregulated carotenoid biosynthesis-related genes—phytoene synthase (*Psy*), lycopene-β-cyclase (*Lcyb*), β-carotene hydroxylase (*CrtZ*), and capsanthin/capsorubin synthase (*Ccs*)—during the experiment. While the expression of the *Ccs* gene was reduced, there was a 5.5-fold upregulation of the *Psy* gene at the end of incubation. At 20 °C, the *Psy* gene was downregulated. These observations suggest that the expression of individual genes is temperature-dependent, and these would affect specific carotenoid compounds. The antioxidant capacity (2,2-diphenyl-1-picrylhydrazyl; DPPH and ferric-reducing antioxidant power; FRAP) values had no difference between temperatures; the higher content of total phenolics and vitamin C presented in the chili at 30 °C probably corresponds to the advanced ripening process. Thus, 30 °C is the recommended incubation temperature for mature green chili to achieve the industry-demanded intense red color and high accumulation of phytochemicals.

## 1. Introduction

The “Takanotsume” chili is a widely used variety in Japanese cuisine, particularly in the making of red spices. At the red ripe stage, this chili has high phytochemicals, including carotenoids, capsaicinoids, polyphenols, and ascorbic acid, containing antioxidant properties [1]. An increase in intense red color and phytochemicals are important qualities for processing the chili. However, one of the major problems for this chili is its harvesting periods. It is commonly harvested at mature green, breaker, and red stages simultaneously [2], leading to an uneven red color after drying [3,4]. This characteristic significantly contributes to a loss of market value. 

**Figure 1:**
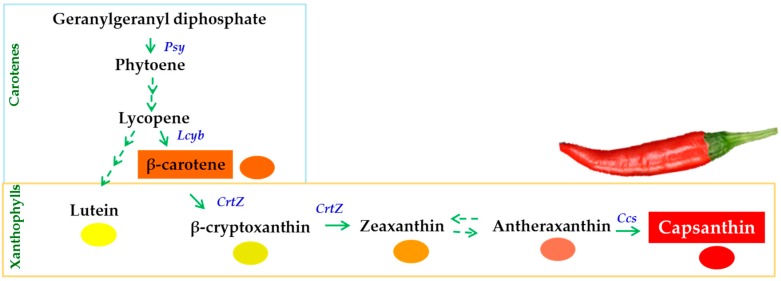
The carotenoid biosynthetic pathway and the key carotenoid biosynthesis-related genes, namely, phytoene synthase (*Psy*), lycopene-β-cyclase (*Lcyb*), β-carotene hydroxylase (*CrtZ*), and capsanthin/capsorubin synthase (*Ccs*), during ripening of *Capsicum* fruit.

In most cultivars of *C. annuum*, the accumulation of chlorophylls declines and that of carotenoids increases during the ripening process [5,6]. Carotenoids are the dominant pigment, and capsanthin compounds contribute up to 50% of the total carotenoids in *C. annuum* [4,7]. These carotenoids have excellent scavenging activity for reactive oxygen species (ROS) [8]. The generation of capsanthin is controlled by key genes—phytoene synthase (*Psy*), lycopene-β-cyclase (*Lcyb*), β-carotene hydroxylase (*CrtZ*), and capsanthin/capsorubin synthase (*Ccs*)—in the carotenoid biosynthetic pathway (Figure 1) [9,10]. Even the ripening processes in *C. annuum* are under genetic control, although the rate of these processes is also influenced by environmental factors. 

The postharvest treatment for inducing an intense red color in red chili, particularly under optimum temperature, is a simple method that is of interest to this investigation. As proposed previously by Acedo [2] and Márkus et al. [4], storage at 20 to 23 °C improved the development of red color in partially-red bell peppers and “Km-622” paprika. However, it took a few weeks for them to achieve a uniform intense red appearance. Nevertheless, an optimum temperature inducing a red coloration of *C. annuum* is not well investigated to date. Different temperature levels have been reported to induce the ripening process and red color in different plant species. In the skin of mango [11], storage under moderately high temperature (30 ± 2 °C) rapidly advanced the ripening processes and increased chlorophyll degradation and total carotenoid accumulation compared with cooler conditions. On the other hand, Matsumoto et al. [12] reported that a postharvest temperature of 20 °C promoted the accumulation of key xanthophylls as β-cryptoxanthin in citrus, whereas a temperature level of either 5 or 30 °C provided a lesser content of this xanthophyll. The same trend was observed in tomato in terms of lycopene accumulation [13]. However, an increase in individual carotenoids, such as zeaxanthin and β-carotene, was observed in citrus and tomato, respectively, when these fruits were stored at a warmer temperature of 30 ± 2 °C. Notably, there is some evidence that the accumulation of individual carotenoids differs with the specific postharvest temperature and plant species. As of now, these specific responses of individual carotenoids to distinct temperature levels in different plant species, including chili, are not well understood. 

Additionally, there is a report that ripening processes, such as chlorophyll breakdown and carotenoid accumulation, in horticultural fruit are hastened under temperatures beyond the optimum condition [14]. These processes would trigger an accumulation of ROS [15]. To balance the amount of ROS, there would be a significant increase in the production of antioxidant compounds such as carotenoids, polyphenols, and ascorbic acid [8,15]. However, the temperature threshold related to the stimulation and/or elimination of phytochemical accumulation and antioxidant capacity in *Capsicum* is not well understood to date. Therefore, the effect of temperature on the formation of red color, expression of carotenoid biosynthesis-related genes, accumulation of phytochemicals, and emergence of antioxidant capacity in harvested mature green “Takanotsume” chili is investigated in this study.

## 2. Materials and Methods

### 2.1. Plant Materials and Experimental Conditions

Seedlings of “Takanotsume” chili were grown at the Tsukuba-Plant Innovation Research Center (T-PIRC) farm, University of Tsukuba, Japan, in 2017. The temperature and relative humidity (RH) in the greenhouse were 28 ± 3 °C and 75 ± 2%, respectively, measured using a data logger (TR-74Ui, T&D Cor., Nagano, Japan). The samples were collected at 32 days after flowering (DAF), and a homogenous color and size were selected for the experiment. Two lots of the samples were detached and incubated at different temperature levels of 20 and 30 °C (75 ± 3% RH) in an incubator (Sanyo MIR-253, SANYO Electric Co., Ltd., Japan) under darkness. The incubated chili was collected on days 0, 1, 2, 3, and 4. The pericarp was frozen in liquid N_2_, grounded into powder, and kept at −80 °C for the subsequent analysis.

### 2.2. Surface Color Analysis

The CIElab (L*, a*, and b*) color space was monitored using a colorimeter (CR-400/410, Konica Minolta, Japan). The L* represents brightness, the a* denotes redness (+)/greenness (−), and the b* displays yellowness (+)/ blueness (−). The chroma means color saturation, which varies from dull to vivid colors (low to high value), and is calculated by ((a*)^2^ + (b*)^2^) ^1/2^. The formula of the hue angle used is tan^−1^ (b*/a*), and ranged from red–purple to blue (0–270°).

### 2.3. Total Phenolic Content (TPC) Determination

TPC was performed following Arnnok et al. [16]. Briefly, 2 g of freeze-dried chili powder was homogenized for extraction containing 20 mL of methanol 90% (*v/v*) mixed with hydrochloric acid 90% (*v/v*) solution (90:10 ratio). It was then stirred for 15 min. The reaction was mixed with 0.2 mL of supernatant, 2.6 mL of distilled water, 2 mL sodium carbonate 7% (*w/v*), and 0.2 mL of Folin-Ciocalteu reagent for TCP quantitative analysis. Following the incubation at 30 °C for 90 min, the absorbance of the reaction mixture was measured at 745 nm (UV/VIS, JASCO Corporation, Tokyo, Japan). The calibration curve of gallic acid (20−100 mg L^−1^) was used for comparing the values obtained and expressed as milligram gallic acid equivalents per gram dry weight of the samples (mg GAE g ^−1^ dw).

### 2.4. Vitamin C Content Determination

The method was carried out as described by Roe et al. [17]. The freeze-dried sample powder (50 mg) was extracted with 4 mL of metaphoric acid 5% (*v/v*). The suspension was centrifuged at 10,000 rpm for 15 min at 4 °C. The reaction mixture was contained 0.4 mL of extract solution, 0.2 mL of indophenol 0.02% (*v/v*), 0.4 mL of thiourea 2% (*v/v*), and 0.2 mL of 2,4-dinitrophenyl hydrazine 2% (*v/v*). Thereafter, the mixture was incubated at 37 °C for 180 min. The reaction was terminated by adding 1 mL of sulfuric acid 85% (*v/v*) and left to stand at 25 °C for 30 min. The absorbance was recorded at 540 nm and the value was expressed as mg g^−1^ dw of *L*-ascorbic acid.

### 2.5. Antioxidant Capacity Determination

The extraction of antioxidant capacity determination was followed from the method of Arslan and Özcan [18]. The freeze-dried sample powder (50 mg) was extracted with 5 mL of methanol 80% (*v/v*) through sonication (Branson 8510, Emerson Corporation, Kanagawa, Japan) for 30 min and then centrifuged at 10,000 rpm for 15 min. The supernatant collected was filtered (Advantec, No. 1) and the filtrate was used for further analysis. 

#### 2.5.1. The 2,2-Diphenyl-1-Picrylhydrazyl (DPPH) Assay

The protocol was based on Arslan and Özcan [18]. The supernatant (0.1 mL) was mixed with 5 mL of 0.1 mM DPPH solution and left to stand for 40 min at 20 °C. The absorbance at 517 nm was measured spectrophotometrically for an intensity reduction of violet color. *L*-ascorbic acid was used as an antioxidant standard and presented as mg vitamin C equivalent g^−1^ dw. 

#### 2.5.2. The Ferric-Reducing Antioxidant Power (FRAP) Assay

The FRAP assay was evaluated based on the previous method [19], with minor modification. The FRAP reagent was freshly prepared, which composed of acetate buffer (pH 3.6), TPTZ (10 mM 2,4,6-tripyridyl-s-triazine in 40 mM hydrochloric acid), and 20 mM iron(III) chloride hexahydrate in a ratio of 10:1:1. The supernatant (0.2 mL) of the sample was added with 2.8 mL FRAP reagent. The reaction mixture was incubated for 30 min without light. The absorbance was read at 593 nm using the spectrophotometer. The antioxidant capacity of the sample was compared to *L*-ascorbic acid and expressed as mg vitamin C equivalent g^−1^ dw.

### 2.6. Pigments Determination

#### 2.6.1. Total Chlorophyll Content

Total chlorophyll was determined using *N,N* dimethylformamide for extraction following Moran [20]. The maximum absorbance of chlorophyll *a* and *b* was measured at 664 and 647 nm, respectively, with the spectrophotometer. The total chlorophyll was calculated from the values of chlorophyll *a* and *b*, which was expressed as μg g^−1^ dw of chili.

#### 2.6.2. Carotenoid Content 

The extraction of carotenoids was performed under the dark condition following Levy et al. [7]. The freeze-dried chili powder (500 μg) was added with 10 mL of acetone consisting of butylated hydroxytoluene (BHT) 0.5% (*w/v*), and 0.2 mL of all-E-β-apo-8′-carotenal (50 µg mL^−1^), used as an internal standard. The suspension was vigorously shaken for 1 min using a vortex before sonicating for 1 h. It was then filtered through a filter paper (Advantec No. 2). The filtrate (5 mL) was evaporated (Buchi, R-3 rotary evaporator fitted with V-700 vacuum pump, California, USA) at 35 °C. The crude extract was solubilized in 3 mL of mobile phase B (methanol:acetonitrile:isopropanol (10:35:55) containing of BHT). The solubilized sample was filtered through a 0.45 µm filter (MillexR-LH, Japan) before injection onto a high performance liquid chromatograph (HPLC) (JASCO Corporation). The operation system of HPLC was performed based on a previous study [9]. Carotenoids were analyzed through a reverse-phase C_18_ column (5 µm, 4.6 × 150 mm; J-Pak Symphonia, JASCO Corporation). The eluent was 9% (*v/v*) water in methanol (A), 10% (*v/v*) methanol:35% (*v/v*) acetonitrile:55% (*v/v*) isopropanol (B), and methanol (C). The operation program of mobile phases was set up according to our previously reported conditions [9]. The gradient program for chemical isolation was first carried out for 20 min using solvents A, B, and C (80%, 0%, and 20%, respectively), and subsequently performed at 0%, 90%, and 10% for 20 to 30 min. The column was then cleaned for 30 min, which run at 80%, 0%, and 20%, respectively. The wavelength employed was 436, 454, and 470 nm in the PDA detector [21]. The preparation of each standard stock was performed as previously described [9]. The free-capsanthin, β-carotene, and all-E-β-apo-8′-carotenal were solubilized in mobile phase B and they were used for standard curves analysis. For the calculation of total carotenoids, all peak areas in the chromatogram (see Figures S1 and S2) were added including the free-, mono- and di-esterified carotenoids, which were compared to the standard curve of free-capsanthin. Each carotenoid content was represented as μg g^−1^ dw of chili.

### 2.7. Carotenoid Biosynthesis-Related Genes Expression Analysis

The RNeasy Mini Kit was used for extracting total RNA based on the manufacturer’s instructions (Qiagen, Hilden, Germany). The concentration of each RNA sample was diluted to 1 μg using RNase-free water. The synthesizing complementary DNA (cDNA) was followed the manufacturer’s instructions (Invitrogen, USA). The expression of *Psy, Lcyb, CrtZ,* and *Ccs* genes in the carotenoid biosynthetic pathway was evaluated by quantitative real-time PCR (qRT-PCR) (M×3000P QPCR instrument, Agilent, USA). The final reaction mixture (10 μL) consisted of 1 μL of cDNA, 5 μL of SYBR Green QPCR Master Mix (Agilent), 0.1 μL of reference dye (ROX), 0.4 of 10 μM primers, and 3.5 μL of RNase-free water. The thermal conditions for setting qRT-PCR instrument have been previously reported [9]. After reheating, the amplified products at 95 °C for 1 min, then at 55 °C for 30 s, and 95 °C for 30 s estimated the melting curves. A single dissociation curve was used to evaluate the specificity of the amplification. The sets of *Psy, Lcyb, CrtZ,* and *Ccs* primers were purchased from Hokkaido System Science Co., Ltd., Japan (see Table S1).

### 2.8. Statistical Analysis

The experiment was conducted in a completely randomized design (CRD). Three replicates were employed in each treatment. The data were subjected to two-sample *t*-test for the analysis of means using the Statistical Package for Social Sciences application program (SPSS Inc., Version 17.0, Chicago, IL, USA). 

## 3. Results

**Figure 2:**
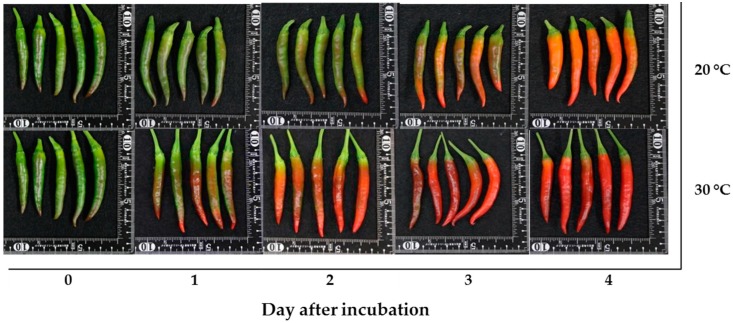
The appearance of “Takanotsume” chili fruit incubated at 20 and 30 °C for 4 days.

The mature green “Takanotsume” chili incubated at 20 and 30 °C was investigated. The results showed that the chili incubated at 30 °C developed an intense red color earlier than that at 20 °C (Figure 2). On day 2 of incubation, the chili incubated at 30 °C had a more intense red color, while the surface of the chili incubated at 20 °C remained color green for more than 90% of the fruit and, then, its color changed to orange–red on day 4 of incubation.

**Figure 3:**
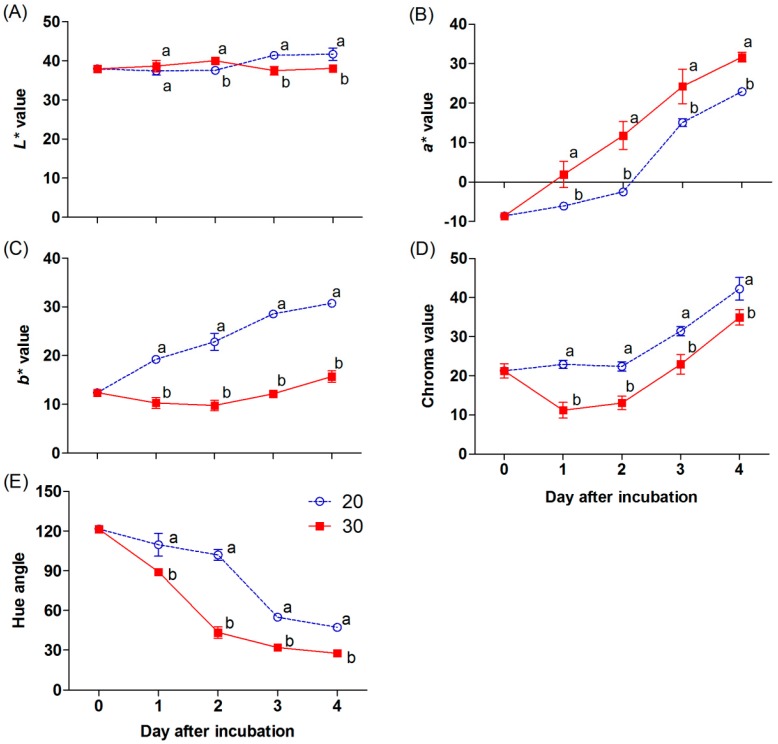
Effect of incubation temperatures at 20 and 30 °C on the L*, a*, b*, chroma, and hue angle (**A−E**) of “Takanotsume” chili for 4 days. Values are means ± SD (*n* = 3). Different letters indicate a significant difference between temperatures on the same day of incubation (*p* ≤ 0.05).

The color appearances of the chili are related to the surface color values (L*, a*, b*, chroma, and hue angle), as shown in Figure 3A to 3E. The L* value slightly increased in the chili at 30 °C, being a significantly higher value on day 2 and declining afterwards. In contrast, the L* value was constant in the chili at 20 °C during the first two days; thereafter, it increased and was significantly higher than that at 30 °C (Figure 3A). The a* value progressively increased, and a significantly higher value was shown in the chili at 30 °C during incubation (Figure 3B). In contrast, higher b* and chroma values were found in the sample incubated at 20 °C (Figure 3C,D). A significant decrease in the hue angle was presented in the chili incubated at 30 °C through the incubation periods (Figure 3E).

**Figure 4:**
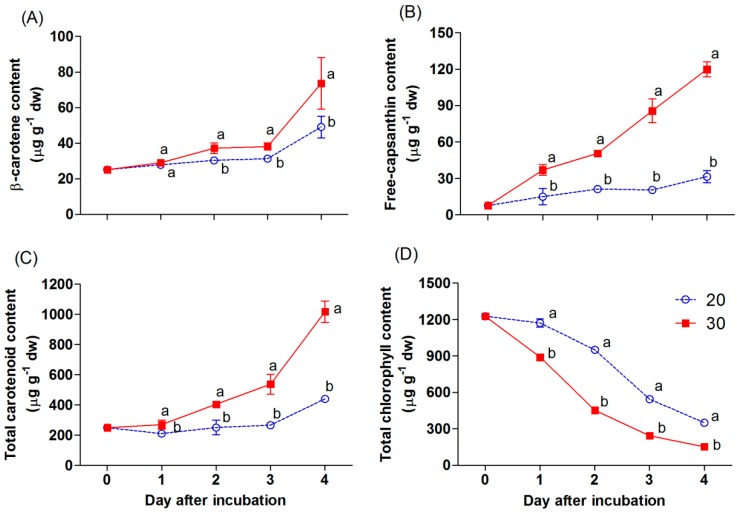
Effect of incubation temperatures at 20 and 30 °C on β-carotene (**A**), free-capsanthin (**B**), total carotenoid content (**C**), and total chlorophyll content (**D**) of “Takanotsume” chili for 4 days. Values are means ± SD (*n* = 3). Different letters indicate a significant difference between temperatures on the same day of incubation (*p* ≤ 0.05).

The appearance of red color in the chili was mainly linked to the accumulation of carotenoid and chlorophyll compounds, as shown in Figure 4A–D. Total chlorophyll declined gradually during incubation (Figure 4D). At 20 °C, total chlorophyll in the chili was maintained during the first day, and decreased afterward, while the content of this pigment in the chili at 30 °C was markedly reduced and had a significantly lower content compared with that at 20 °C. On the other hand, the accumulation of carotenoids progressively increased in both treatments during incubation (Figure 4A−C). The free-capsanthin content was significantly higher in the chili incubated at 30 °C and increased by a 3.8-fold difference with the content of the chili at 20 °C on day 4 of incubation (Figure 4B). The β-carotene content in the chili at 30 °C was slightly increased during the first three days of incubation (Figure 4A). Afterward, it markedly increased in all samples. The β-carotene content was significantly higher in the sample incubated at 30 °C after day 2 of incubation. The total carotenoid content also increased throughout (Figure 4C). The results showed a similar trend with the accumulation of free-capsanthin and β-carotene.

**Figure 5:**
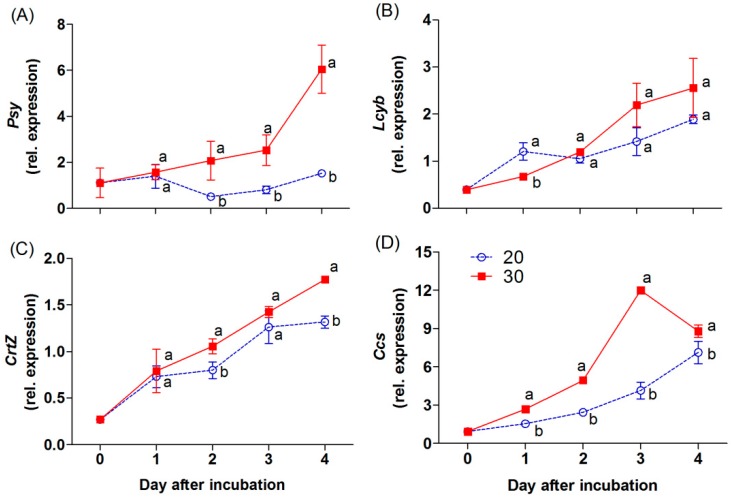
Effect of incubation temperatures at 20 and 30 °C on the relative expression levels of *Psy* (**A**), *Lcyb* (**B**), *CrtZ* (**C**), and *Ccs* (**D**) of “Takanotsume” chili for 4 days. Values are means ± SD (*n* = 3). Different letters indicate a significant difference between temperatures on the same day of incubation (*p* ≤ 0.05).

The *Psy, Lcyb, CrtZ*, and *Ccs* genes are the key genes in the carotenoid biosynthetic pathway of the chili (Figure 1). The expression level of the *Psy* gene decreased during incubation at 20 °C. On the other hand, an overexpression by 5.5-fold, relative to the initial day of the *Psy* gene, was found in the chili at 30 °C on day 4 of incubation (Figure 5A). At 30 °C, the *Ccs* gene was markedly upregulated during the first three days; thereafter, its expression level decreased at the end of the experiment, but this gene was higher than at 20 °C (Figure 5D). The expression level of the *Lcyb* gene progressively increased at both incubation treatments (Figure 5B). Although the expression of this gene did not show a significant difference between the temperatures, the higher trend was found in the chili at 30 °C. The expression level of the *CrtZ* gene also increased gradually, and a significant increase was found in the chili at 30 °C on days 2 and 4 of incubation (Figure 5C).

**Figure 6:**
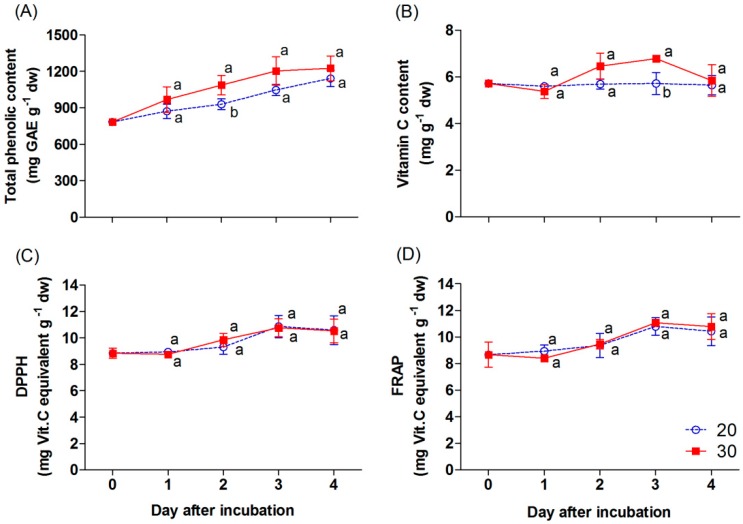
Effect of incubation temperatures at 20 and 30 °C on total phenolic content (**A**), vitamin C content (**B**), and antioxidant capacity values; DPPH (**C**) and FRAP (**D**) of “Takanotsume” chili for 4 days. Values are means ± SD (*n* = 3). Different letters indicate a significant difference between temperatures on the same day of incubation (*p* ≤ 0.05).

The TPC in the samples progressively increased during incubation, and the TPC in the chili incubated at 30 °C was significantly higher compared with 20 °C, particularly on day 2 of incubation (Figure 6A). The vitamin C content remained unchanged in the chili at 20 °C (Figure 6B), while it increased in the sample at 30 °C on days 2 and 3 of incubation. The content slightly decreased thereafter and achieved almost the same value as chili at 20 and 30 °C on day 4. The antioxidant capacity, as indicated by the DPPH and FRAP values, slightly increased through incubation (Figure 6C,D). However, there were no significant differences in the value of both evaluated antioxidants between the temperature treatments.

## 4. Discussion

An increase in the uniformity of the red color in chili is a critical factor before processing. Naturally, the appearance of the red color in chili is controlled by internal and external factors [22]. One of the main environmental stimulants is temperature, which plays a crucial role in color development and the superficial appearance of fruits [23]. In mature green “Takanotsume” chili, rapid red coloration was observed during incubation at 30 °C compared with that at 20 °C (Figure 2). This occurrence was correlated with a higher a* and lower L*, b*, and hue angle by the second day (Figure 3A−E).

Generally, the appearance of the red color in red chili is linked to an accumulation of carotenoids, namely, red xanthophylls. The major red xanthophyll in red chili peppers is capsanthin [24]. Furthermore, one of the dominant carotenes in red chili is β-carotene, which is an important intermediate in synthesizing the red xanthophylls [7,25]. The concentration of those pigments normally increases as the chili achieves ripening [4,25]. In this study, the incubation temperature levels impacted the accumulation of these carotenoids in this chili. A significant increase in the accumulation of β-carotene and free-capsanthin was found in the sample incubated at 30 °C compared with that at 20 °C (Figure 4A,B). Thus, the higher temperature of 30 °C hastened the intense red appearance in the mature green “Takanotsume” chili within two days. In contrast, the total chlorophyll was gradually reduced during incubation (Figure 4D). A loss of total chlorophyll was stimulated in the chili at 30 °C throughout the experiment. This indicates that the degradation of chlorophylls in this chili is stimulated at high temperature. The chlorophylls are known to decrease during ripening in the chili [19]. At the onset of ripening in most varieties of chili, chlorophylls markedly decrease, whereas carotenoids increase as chloroplasts are transformed into chromoplasts, leading to the appearance of red color [5,6]. However, these biological processes are controlled genetically and can be accelerated by moderately high temperatures. The same trend was observed in other fruits [26], such as in the mango skin, wherein early chlorophyll disappearance was found in fruit stored at 28 to 32 °C compared with that at a lower temperature (7–20 °C) [11].

An increase in the accumulation of carotenoids in chili was attributed to the expression of carotenoid biosynthesis-related genes, including *Psy*, *Lcyb*, *CrtZ*, and *Ccs* (Figure 5A−D). All genes progressively increased throughout, except the expression of the *Psy* and *Ccs* genes, which depend on the incubation temperature and duration. The *Psy* gene was downregulated by 2.1-fold in the chili, relative to day 0 at 20 °C. However, this gene was upregulated at 30 °C, whereas the *Ccs* gene showed a negative response at 30 °C on day 4 of the experiment. This indicates that temperature influenced the expression of tested genes, particularly the *Psy* and *Ccs* genes. Thus, lesser amounts of β-carotene, free-capsanthin, and total carotenoids were observed at 20 °C (Figure 4A−C), which caused the red–orange color on the last day of incubation (Figure 2). In contrast, the chili at 30 °C presented an intense red color, caused by the higher expression of all tested genes. The results imply that *Psy* and *Ccs* are the critical genes that regulate carotenoid biosynthesis at different temperature levels, affecting the surface color in “Takanotsume” chili.

At 30 °C, the expression of the *Ccs* gene decreased at the end of the experiment, but it did not affect the increase in the accumulation of free-capsanthin after incubation at 30 °C for 4 days (Figure 4B). This phenomenon may be related to the rapid transformation of precursors into capsanthin during the first three days due to high temperature. When the full red color stage was achieved in the chili, the *Ccs* gene perhaps did not increase in expression during this maturity stage. On the other hand, the overexpression of the *Psy* gene at 30 °C would result in the generation of the intermediate compounds in the carotenoid biosynthetic pathway. This would probably receive more antioxidant compounds to cope up with the oxidative stress produced [15], in addition to the capsanthin, during the ripening processes of the chili.

Originally, *C. annuum*, including the “Takanotsume” chili, is found in tropical regions [1,23]. It requires temperatures between 21 and 30 °C throughout its developmental cycle, including color development either pre- or post-harvest [27]. Thus, the incubation at 30 °C would be an ideal temperature for inducing the main carotenoid accumulation and rapid intense red coloration by the second day of incubation for this chili. In other tropical fruits, Thomus and Janave [11] illustrated that accumulation of total carotenoids in mango skin increased at 30 ± 2 °C, whereas lower temperatures between 7 and 20 °C reduced this pigment. Therefore, in tropical plants, including the “Takanotsume” chili, greater relatively warm temperature at 30 ± 2 °C hastens carotenoid accumulation. However, the optimum temperature for incubation requires more investigation to understand the temperature threshold related to the acceleration and/or suppression of the ripening processes in chili.

Notably, the exact regulation of carotenoid biosynthesis in *Capsicum* under high temperatures is not well understood. From our results, the incubation temperature at 30 °C was more effective in inducing the expression of all determined genes compared with 20 °C. This is different from experiments with citrus, where the enhancement of individual carotenoids in the flavedo was shown during storage at 5, 20, and 30 °C [12]. At 20 °C, the major carotenoid, β-cryptoxanthin, in the flavedo was markedly increased, whereas this compound was lower during storage at both 5 and 30 °C, except for zeaxanthin, whose highest content was found at 30 °C. The same trend was documented in tomato by Gautier et al. [13], where high lycopene content and rapid red coloration in the fruit occurred between 21 and 26 °C, whereas an increasing temperature of 27 and 32 °C reduced lycopene but increased the β-carotene content. These studies indicate that the optimum temperature for enhancing the main carotenoids in citrus and tomato as sub-tropical fruits is a relatively low temperature, between 20 and 26 °C. However, some individual carotenoids, such as zeaxanthin in citrus and β-carotene in tomato, increased at the higher temperature of 30 ± 2 °C compared with the temperature of 20 to 26 °C. Furthermore, zeaxanthin and violaxanthin were stimulated in leaves of tobacco and *Arabidopsis* when they were exposed to 40 °C compared with 23 °C [28]. This probably implies the increase in expression of the *Psy* gene (Figure 5A) and the decrease in the *Ccs* gene (Figure 5D) on the fourth day of our experiment results in different individual carotenoid accumulation in chili. Notably, the specific temperature level that induces the accumulation of individual carotenoids in plant species influencing the biosynthesis pathway is still unclear.

As noted by an earlier study, improper temperature levels would stimulate biochemical reactions [15]. The high temperature of 30 ± 2 °C enhances ripening processes, as well as respiration in fruit, with mango as an example [11]. These biological processes would cause oxidative stress and an accumulation of ROS. The plant balances this condition with antioxidant systems to defend the products from this stress [29]. The same trend has been found in tomato [13], that is, a high temperature of 27 to 30 °C increased the total phenolic content compared with a slightly lower temperature (21−26 °C). Tan et al. [30] and Sun et al. [31] reported that polyphenols are one of the important antioxidative properties that generally increase during chili fruit ripening either pre- or post-harvest. This is similar to the findings of our study, where the rapid ripening processes would increase the production of ROS in the mature green “Takanotsume” chili at 30 °C. Therefore, the significant increase in total phenolic content was shown in the chili incubated at 30 °C throughout the incubation time compared with that of 20 °C (Figure 6A).

Generally, vitamin C also plays an antioxidant role in plants. In our study, the vitamin C content was slightly increased in the chili incubated at 30 °C on day 3; afterward, it decreased and had no significant difference compared with that at 20 °C. The vitamin C content remained unchanged for the chili incubated at 20 °C (Figure 6B). The ascorbate content involved in the response against temperature changes had also been shown in a prior report in pre-harvested sweet pepper fruits. The higher decreased ascorbate content was shown in pepper growth at a higher temperature compared with those developed in colder conditions [32]. Thomus and Janave [11] documented that ascorbic acid levels in mango were progressively reduced during storage at 30 ± 2 °C for 13 days, while the storage at a lower temperature (7–20 °C) revealed an increase during 28 days of storage. At 30 °C, the decrease in vitamin C content was observed on day 4 of incubation, perhaps due to its antioxidant function through the ripening processes with its less production (Figure 6B). On the other hand, the gradual increase of carotenoid contents would be the other antioxidant to cope with the oxidative stress on the fourth day of the experiment in the chili incubated at 30 °C (Figure 4A−C ). Therefore, TPC and vitamin C, as well as the carotenoid content as an antioxidative stress function, increased during incubation at a higher temperature (30 °C) throughout the ripening process.

The antioxidant capacity in the chili, indicated by the DPPH and FRAP values, was slightly increased during incubation. However, the antioxidant capacity of the chili showed no significant difference between the temperature at 20 and 30 °C (Figure 6C,D). The antioxidant capacity had the same trend as TPC (Figure 6A). Similarly, Tan et al. [30] had documented that the antioxidant capacity in “Kulai” pepper was strongly associated with the increasing total phenolics. It is noteworthy that even the chili incubated at 20 °C had the lower TPC, but the vitamin C content showed no difference between the treatments (Figure 6B). Thus, the scavenging of ROS in the chili incubated at 20 °C was probably due to the functions of vitamin C. Nevertheless, there are many phytochemicals present in *C. annuum* that function as antioxidants, and this point requires further study.

## 5. Conclusions

The changes in color through the loss of chlorophylls and carotenoids accumulation in mature green “Takanotsume” chili incubated at 20 and 30 °C were observed. The gradual increase in β-carotene and free-capsanthin under 30 °C was attributed to the upregulation of the *Psy, Lcyb, CrtZ,* and *Ccs* genes, except the *Ccs* gene, which was downregulated slightly in the last 4 days of incubation. Contradicting results were shown at 20 °C, with lesser amounts of carotenoids due to the downregulation of the *Psy* gene compared with the initial level and the lower expression of all tested genes. Results suggest that the response of carotenoid biosynthesis-related genes is regulated by specific temperature levels providing carotenoid accumulations differently. However, the temperature threshold related to the acceleration and/or suppression of ripening processes at the molecular level needs to be better understood. We also observed that the high temperature at 30 °C induced the accumulation of total phenolics and vitamin C, which plays an important function in dealing with oxidative stress during the advancement of ripening processes. For the industrial processing of “Takanotsume” chili harvested in the unripe stage, the post-harvest temperature at 30 °C is recommended to speed up the appearance of red color via a high accumulation of carotenoids, as well as, total phenolics and vitamin C content.

## Supplementary Materials

The following are available online at https://www.mdpi.com/2076-3921/9/3/203/s1, Table S1: The sets of primer sequences of *Psy*, *Lcyb*, *CrtZ*, and *Ccs* used for the qRT-PCR of the carotenoid biosynthesis-related genes. Figure S1: Chromatogram of carotenoid profiles in “Takanotsume” chili after harvested at fully red stage fruit and mature green stage. Number 1 and 2 in the chromatogram of fully red fruit were expected to be the mono- and di-esterified carotenoid forms. Figure S2: Chromatogram of carotenoid profiles in “Takanotsume” chili after incubation at 20 and 30 °C for 4 days.
