# Supplementary material for: Influence of Postharvest Temperatures on Carotenoid Biosynthesis and Phytochemicals in Mature Green Chili (Capsicum annuum L.)
Source: Antioxidants (Basel). 2020 Mar 1;9(3):203. doi: 10.3390/antiox9030203 (PMC7139383; doi:10.3390/antiox9030203)
Supplement: Supplementary file 1 [file antioxidants-09-00203-s001.pdf]

Supplementary Materials

Table S1. The sets of primer sequences of *Psy*, *Lcyb*, *Crt*Z, and *Ccs* used for the qRT-PCR of the carotenoid biosynthesis-related genes.

**GenBank/Genes**	**Forward primer(5′→3′)**	**Reverse primer(5′→3′)**
X68017.1/ Phytoene synthase; *Psy*	TCTCAAACGGGACAGGATTC	CCACCTTTGTTTTCCACCTC
X86221.1/ Lycopene-β-cyclase; *Lcyb*	AGTTATCCGCAGCTGTTTGG	CCATGCCAATAACGAGGTTC
Y09225.1/ β-carotene hydroxylase; *CrtZ*	GCATATGCACGAGTCACACC	GGGATAAGGCCTTTATGGTT
X76165.1/Capsanthin/capsorubin synthase; *Ccs*	TCGAAAGCCTTGGCTCAACA	GAAAGGAACCCGTGCCAGTA
AY486137.1/ Ubiquitin conjugating; *Ubi3*	TGTCCATCTGCTCTCTGTTG	CACCCCAAGCACAATAAGAC

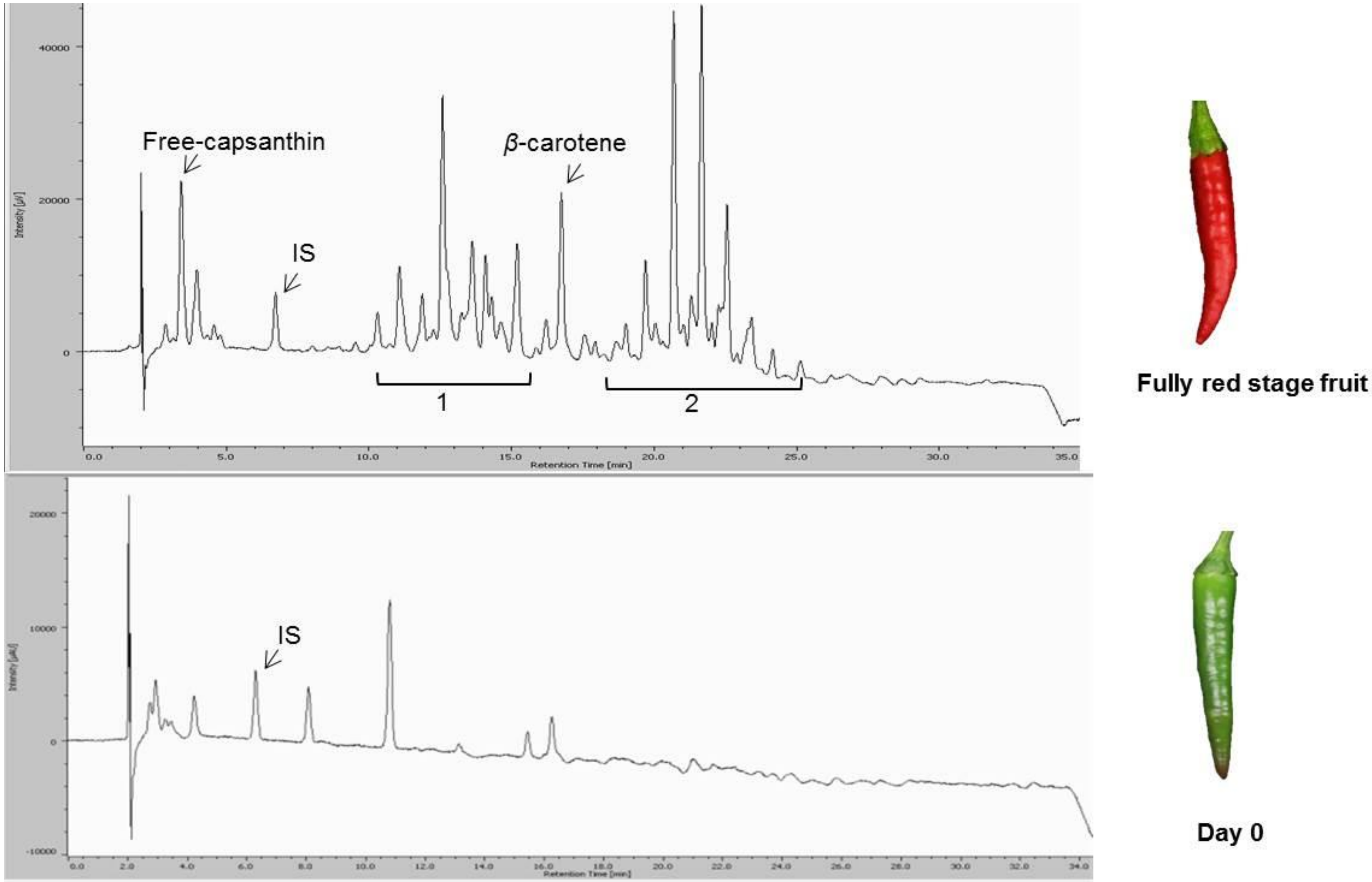

Figure S1. Chromatogram of carotenoid profiles in "Takanotsume" chili after harvested at fully red stage fruit and mature green stage. Number 1 and 2 in the chromatogram of fully red fruit were expected to be the mono- and di-esterified carotenoid forms.

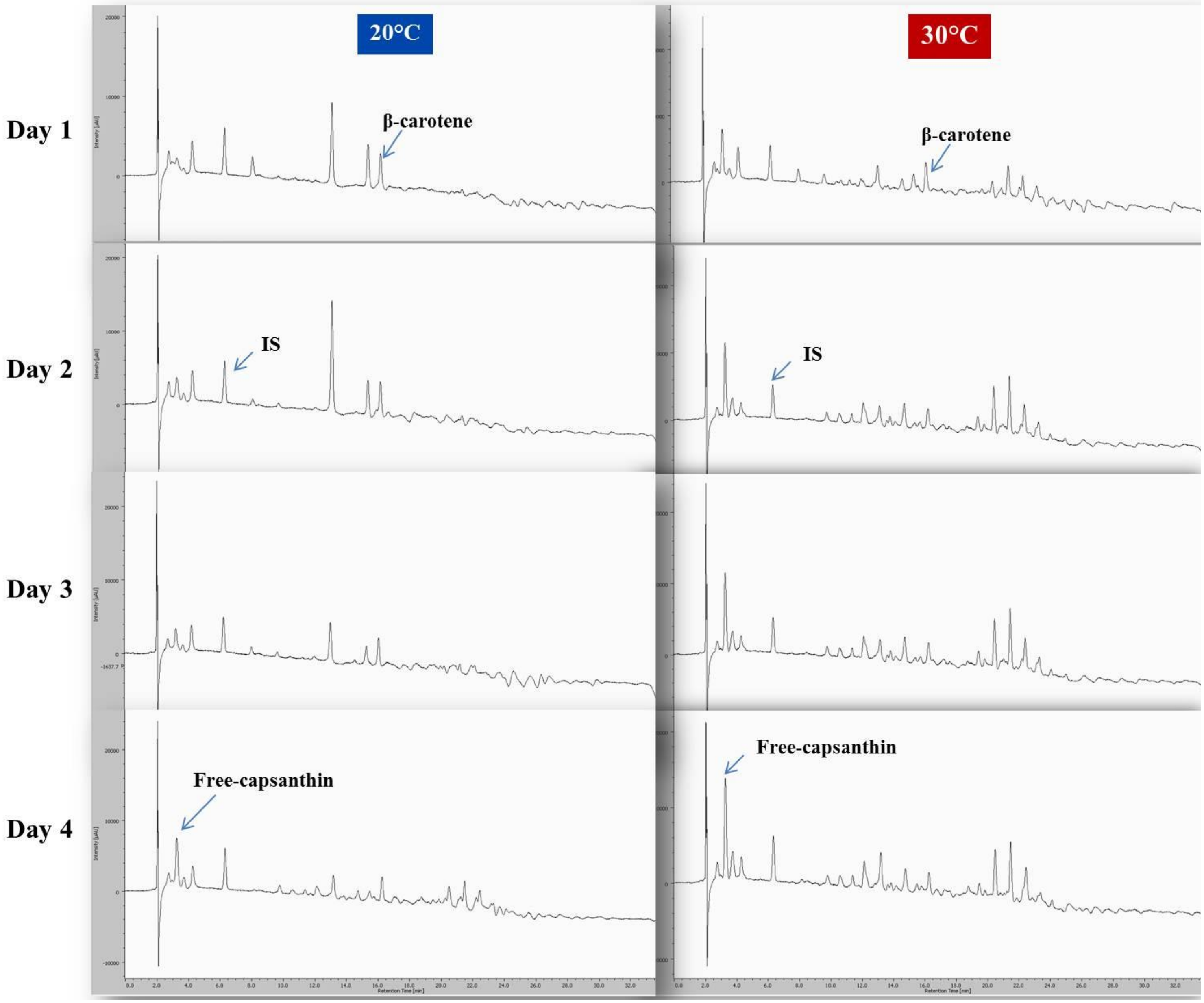

Figure S2. Chromatogram of carotenoid profiles in "Takanotsume" chili after incubation at 20 °C and 30 °C for 4 days.
